# Impact of the COVID-19 lockdown on lifestyle behaviors and their association with personality among adults in Qatar: A cross-sectional study

**DOI:** 10.1371/journal.pone.0276426

**Published:** 2022-11-11

**Authors:** Tamara Al-Abdi, Alexandros Heraclides, Alexia Papageorgiou, Elena Philippou

**Affiliations:** 1 Department of Human Nutrition, Qatar University, Doha, Qatar; 2 Department of Health Sciences, School of Sciences, European University Cyprus, Nicosia, Cyprus; 3 Department of Basic and Clinical Sciences, University of Nicosia Medical School, Nicosia, Cyprus; 4 Department of Life and Health Sciences, School of Sciences and Engineering, University of Nicosia, Nicosia, Cyprus; 5 Department of Nutritional Sciences, King’s College London, London, United Kingdom; St John’s University, UNITED STATES

## Abstract

The coronavirus pandemic (COVID-19) resulted in lockdowns and social distancing measures enforced by governments around the world. This study aimed to identify changes in adherence to the Mediterranean diet (MD) and physical activity (PA) and associations with personality during lockdown. Using a cross-sectional design, a convenient sample of 543 adults in Qatar completed an online questionnaire consisting of validated tools to measure adherence to MD (MEDAS questionnaire, score ranges 0–13), PA (IPAQ, assessing light, moderate high intensity PA) and personality (BFI-10, categorizing individuals’ personalities). The majority of the participants were female (89%), aged between 21 and 29 years (45%). The overall MD adherence decreased during lockdown (5.9 vs. 6.1, p < 0.001). There was an increase in olive oil (9% vs. 12%; p < 0.001), vegetables (54.3% vs. 58.7%; p = 0.005), legumes (11.8% vs. 15.3%; p = 0.007), sofrito (70.9% vs. 77.3%; p < 0.001) and fat (45.9% vs. 53.8%; p < 0.001) consumption and a decrease in fresh fruit (39.4% vs. 15.8%; p < 0.001) and fish/seafood (5.9% vs. 3.9%; p = 0.0035) consumption during lockdown. Met-min/week values of total PA (1330.5 vs. 1836.7), vigorous activity (711.5 vs. 867.4), moderate activity (208.3 vs. 301.3), and walking (410.7 vs. 668.0) all decreased during lockdown (p < 0.001, p = 0.010, p = 0.010 p < 0.001, respectively), while sitting increased (3837.3 vs. 2896.4 p < 0.001). The extraversion personality dimension had a higher MD adherence (p = 0.039) compared to agreeableness before lockdown. No changes in MEDAS scores were observed during lockdown in those with high levels of openness. Openness was positively associated with all PA (*p* = 0.027), including walking (*p* = 0.026), and negatively associated with sitting (*p* = 0.038) before lockdown, while participants with high scores for neuroticism were less likely to be sitting during lockdown (*p* = 0.042). The findings can be used to guide the development of appropriate personality-tailored lifestyle interventions.

## Introduction

Coronavirus disease 2019 (COVID-19) was first reported in Wuhan, China, in December 2019, and has since spread all over the globe [1]. On March 11th 2020, the World Health Organization (WHO) declared the outbreak a pandemic [2]. Countries adopted protective measures to avoid infection and to restrict the spread of the virus, which included social distancing, home confinement and the closure of businesses and schools. In Qatar, such lockdown measures were declared on March 9th 2020 [3]. The Qatari population was required to stay at home and only go out for essential needs, while only essential workers were allowed to work with physical distancing guidelines. On June 15th 2020, Qatar lifted lockdown restrictions and slowly reopened in four phases to include parks, shops and restaurants [4].

While these strict preventive measures were considered necessary, they may have changed lifestyle-related daily habits. Home confinement can affect diet, food choice and physical activity patterns [5]. Most studies on the changes in health behaviors during the COVID-19 pandemic in various countries have reported an increased consumption of unhealthy foods, increases in snacking, reduction in fresh food consumption (mainly fruit and fish) and an increase in body weight [6, 7]. On the other hand, some studies have also reported a shift towards a healthier diet with an increase in consumption of fruits and vegetables, fish, legumes and a decrease in fast food consumption [6]. Unhealthy eating habits, an increase in weight gain and sedentary lifestyles were reported in studies within the Gulf region including in Qatar [8, 9]. In contrast, a study by Ben Hassen et al. [3] in Qatar found an increase in fruit and vegetable intake and a decrease in fast food consumption.

The COVID-19 lockdown measures have also resulted in changes in physical activity. The closure of gyms and enforcement of curfews in several countries has resulted in limiting participation in both indoor and outdoor activities. Restrictions have reduced overall physical activity, time, frequency and access to exercise. Several studies have reported a decrease in all levels of physical activity, whether vigorous, moderate or walking during the COVID-19 lockdown, and an increase in sedentary behaviors [10]. However, a few studies have also reported an increase in physical activity [11, 12].

Existing studies have attested to the association between personality traits and health behavior [13–15]. Among the personality traits defined by the five-factor model (FFM), conscientiousness and neuroticism emerge as the most relevant predictors of health behaviors [16]. As recently reviewed [17], individuals who score highly in conscientiousness tend to consume breakfast, have their meals at regular times each day, and exercise more. On the other hand, neuroticism was positively correlated with emotional eating and consumption of sweet foods, eating less fruit and vegetables and physical inactivity [17]. Although it is known that personality traits are stable and tend to be resistant to stressful life events [18], individual differences in personality may contribute to how people respond and behave during a pandemic. These responses may be attributed through beliefs and attitudes associated with the different traits [16].

Limited studies have examined the association between personality traits and health behaviors during the COVID-19 pandemic. Kroencke et al. [19] found that individuals scoring highly in neuroticism experienced a more negative effect in their daily lives during the pandemic. However, Zhang et al. [20] found that the pandemic did not influence the wellbeing of those who scored highly in openness and conscientiousness. In other studies, conscientiousness was associated with higher adherence of containment measures and an increase in physical activity [21], while those with high scores for extraversion were less likely to adhere to physical social distancing measures [22]. People with the latter personality type, however, were more likely to make healthier eating choices during the pandemic [23]. Agreeableness and extraversion were associated with positive changes in physical activity, while neuroticism was associated with a decrease in physical activity levels [24].

The rigorous restrictions put in place in Qatar may have affected dietary intake and exercise habits. Nevertheless, there is limited evidence to evaluate the effect of confinement on these lifestyle factors in association with personality. This is important since personality should be taken into account in the development of both educational programs and policies aiming to address lifestyle during the pandemic or other similar circumstances if these occur. The main aim of this study was thus to investigate for the first time the immediate impact of the COVID-19 lockdown on diet and exercise and the associations with personality and socio- demographics.

## Materials and methods

### Study design and participants

A cross sectional study was conducted from 24^th^ April to 23^rd^ May 2020, during which time Qatar was under the COVID-19 lockdown. Participants were recruited through social media (WhatsApp and Twitter) and by email using a snowball convenience sampling method. Respondents were asked to complete an online questionnaire anonymously that was distributed through social media and encouraged to distribute the questionnaire to their contacts residing in Qatar. The inclusion criteria was adults, aged 18 years or older, who were residents of Qatar during the spring 2020 lockdown. Those not residing in Qatar were excluded. Despite the initial sampling approach, it became apparent that snowball sampling was not followed by the majority of participants, rendering our sample more like a “convenient” sample rather than a “snowball” sample. Participants were asked to provide written informed consent prior to completing the questionnaire. Ethical approval for this study was obtained from Qatar University Institutional Review Board (QU-IRB 1081-EA/19), and it was conducted in accordance with the Declaration of Helsinki.

### Questionnaire

The questionnaire consisted of 78 questions structured into 4 sections: (1) socio demographics, (2) dietary habits before and during the COVID-19 lockdown, (3) physical activity before and during the COVID-19 lockdown, and (4) personality, described in detail below.

#### Socio demographic data

Participants were asked to report their age (≤20 years; 21–29 years; 30–39 years; ≥40 years), gender (male or female), nationality (Arab or non-Arab), highest education level attained (secondary school, university undergraduate, university postgraduate), marital status (single, married, divorced), work status (student, employed, unemployed, retired), smoking status (yes or no), and weight and height during the lockdown. Self-reported data on height and weight were used to calculate BMI (kg/m^2^) and interpreted according to the WHO classification as underweight (BMI: <18.5 kg/m^2^), normal weight (BMI: 18.5–24.9 kg/m^2^), overweight (BMI: 25.0–29.9 kg/m^2^) and obese (BMI: ≥ 30 kg/m^2^).

#### Dietary assessment

Dietary habits were assessed using the MD Adherence Screener (MEDAS) [25], which has been shown to be a valid tool for assessment of MD adherence [26]. The questionnaire comprised 14 questions which assessed the intake of: olive oil as the main fat, tablespoons of olive oil used, vegetables, fruit and fruit juices, meat and meat products, fats (butter, margarine, cream), sweet or carbonated beverages, wine, legumes, fish or shellfish, commercial sweets or pastries, unsalted nuts, poultry and sofrito sauce. Responses that were favorable to the adoption of the MD diet were scored as one point, while responses that were unfavorable were scored as zero. Taking into consideration cultural and religious practices in Qatar, the component related to alcohol (i.e., wine) consumption was not included in the questionnaire, leaving only 13 components of the MEDAS score, and thus the score range of 0 (minimal level of adherence) to 13 (maximum level of adherence). Additionally, ‘ghee’ was added under the question regarding fat consumption since this is commonly used in Qatar.

#### Physical activity assessment

The short version of the International Physical Activity Questionnaire (IPAQ-SF) [27] was used to assess physical activity. IPAQ is a validated tool extensively used to categorize individuals as having low, medium or high PA levels based on the frequency and duration of activities of various intensities and the time spent seated over the past 7 days [28]. The weekly PA level was calculated as energy expenditure in Metabolic Equivalent Task-minutes/week (MET-min/week.), using the assigned MET for each task (3.3 for walking, 4.0 for moderate-intensity PA, and 8.0 for vigorous-intensity PA). Computations of MET-min/week were calculated following the IPAQ recommendations for scoring protocol [27].

#### Personality

Personality traits were assessed using the 44-item validated short version of the Big Five Inventory (BFI-44) [29], which classifies these traits into five broad dimensions: agreeableness, extraversion, conscientiousness, neuroticism and openness based on the five factor model. Participants were asked to report their level of agreement on a 5-point scale ranging from 1—strongly disagree to 5—strongly agree. The BFI-44 is one of the most commonly used instruments in studies about personality, given its reliability and validity in diverse and cross-cultural samples [30]. Participants were asked to report their level of agreement with statements about how they see themselves on a five-point scale ranging from 1 = strongly disagree to 5 = strongly agree. An eight-item scale was used to assess agreeableness (e.g., “I see myself as someone who is helpful and unselfish with others”). A nine-item scale was used to assess extraversion (e.g., “… is outgoing, sociable”). A nine-item scale was used to assess conscientiousness (e.g., “… does a thorough job”). An eight-item scale was used to assess neuroticism (e.g., “… gets nervous easily”). A ten-item scale was used to assess openness (e.g., “… is curious about many different things”). Items were reverse scored where necessary, and the mean was taken across items for each trait, with higher scores indicating a higher observance of the traits.

### Statistical analysis

Distribution of numeric variables was assessed using normality plots. Paired t-tests were used to determine the association between paired numeric variables, the McNemar test for paired binary variables and Bowker’s symmetry test for categorical variables with >2 categories (e.g., MD adherence: low, moderate, high), before and during the COVID-19 lockdown. Association of personality dimensions with dietary habits (MD adherence) and PA were investigated in multiple linear regression models using personality as the main independent variable of interest. No adjustments were made in the first regression model, while the second model was adjusted for gender, age, nationality, education, marital status, employment, smoking and BMI. A power analysis was conducted, which revealed that with a sample of 485 and above we have 80% power to detect a small size effect (d = 0.02) with α error of 0.05, in our linear regression models including nine predictor variables (personality plus 8 covariates).

Descriptive data are presented as number and percentage in parentheses (%) for categorical variables and mean (standard deviation) for continuous variables. Results from the linear regression models represent the mean difference (95% CI) in each numeric outcome of interest (MD adherence and PA) by personality (agreeableness acts as reference category). Results were considered statistically significant if the p-value was < 0.05. Data analysis was performed using STATA (Version 16, Stata Corporation, and College Station, TX, USA).

## Results

### Participants socio demographic characteristics

Six hundred and five online questionnaires were sent out, of which a total of 560 (92.5% response rate) participants responded and completed. Seventeen were excluded due to incomplete data, leaving a final sample of 543 participants. The demographic characteristics of the participants are shown in Table 1. In brief, most of the participants were female (89%) and almost half (45%) were aged between 21 and 29 years. Most were Arab (73.7%), students (46%), held an undergraduate level of education (77.7%), single (63.4%) and nonsmokers (91.9%). Regarding body weight, 31.3% were classified as overweight (BMI = 25.0–29.9 kg/m^2^) and 13.3% were classified as obese (BMI = ≥30 kg/m^2^). With respect to personality, the majority of participants scored highly in openness (41.4%). Females were overrepresented among those scoring highly in agreeableness (95.5%), and males were overrepresented among those scoring highly in conscientiousness (28.6%) (*p* < 0.001). With regards to body weight, those with high scores in agreeableness were more likely to be underweight or normal weight, while those with high scores in openness were more likely to be overweight and obese (*p* < 0.001).

**Table 1:** Characteristics of the studied population by personality during the COVID-19 lockdown.

		Personality Types						
		All	Extraversion	Agreeableness	Conscientiousness	Neuroticism	Openness	*P-value
Variables		(n = 543)	(n = 19)	(n = 179)	(n = 105)	(n = 15)	(n = 225)	
Gender	Female	480 (88.4)	16 (84.2)	171 (95.5)	75 (71.4)	13 (86.7)	205 (91.1)	< 0.001
	Male	63 (11.6)	3 (15.8)	8 (4.5)	30 (28.6)	2 (13.3)	20 (8.9)	
Age (years)	≤ 20	113 (20.8)	3 (15.8)	51 (28.5)	13 (12.4)	1 (6.7)	45 (20.0)	< 0.001
	21–29	243 (44.8)	10 (52.6)	86 (48.0)	33 (31.4)	8 (53.3)	106 (47.1)	
	30–39	99 (18.2)	2 (10.5)	22 (12.3)	28 (26.7)	2 (13.3)	45 (20.0)	
	≥ 40	88 (16.2)	4 (21.1)	20 (11.2)	31 (29.5)	4 (26.7)	29 (12.9)	
Nationality	Non-Arab	143 (26.3)	6 (31.6)	41 (22.9)	41 (39.1)	3 (20.0)	52 (23.1)	0.020
	Arab	400 (73.7)	13 (68.4)	138 (77.1)	64 (61.0)	12 (80.0)	173 (76.9)	
Education	Secondary	28 (5.2)	1 (5.2)	10 (5.6)	2 (1.9)	0 (0.0)	15 (6.7)	0.004
	Undergraduate	422 (77.7)	15 (79)	155 (86.6)	77 (73.3)	13 (867)	162 (72.0)	
	Postgraduate	93 (17.1)	3 (15.8)	14 (7.8)	26 (24.8)	2 (13.3)	48 (21.3)	
Marital Status	Single	344 (63.4)	13 (86.4)	129 (72.1)	41 (39.0)	7 (46.7)	154 (68.4)	< 0.001
	Married	199 (36.7)	6 (31.6)	50 (27.9)	64 (61.0)	8 (53.3)	71 (31.6)	
Work Status	Student	250 (46.0)	9 (47.4)	108 (60.3)	24 (22.9)	4 (26.7)	105 (46.7)	< 0.001
	Employed	76 (14.0)	3 (15.8)	24 (13.4)	11 (10.5)	4 (26.7)	34 (15.1)	
	Unemployed	217 (40.0)	7 (36.8)	47 (26.3)	70 (66.7)	7 (46.7)	86 (38.2)	
Smoker	No	499 (91.9)	17 (89.5)	174 (97.2)	93 (88.6)	14 (93.3)	201 (89.3)	0.033
	Yes	44 (8.1)	2 (10.5)	5 (2.8)	12 (11.4)	1 (6.7)	24 (10.7)	
BMI	Underweight	44 (8.1)	4 (21.0)	15 (8.4)	8 (7.6)	1 (6.7)	16 (7.1)	< 0.001
	Normal	257 (47.3)	12 (63.2)	111 (62.0)	39 (37.2)	8 (53.3)	87 (38.7)	
	Overweight	170 (31.3)	3 (15.8)	39 (21.8)	50 (47.6)	6 (40.0)	72 (32.0)	
	Obese	72 (13.3)	0 (0.0)	14 (7.8)	8 (7.6)	0 (0.0)	50 (22.2)	

Statistical significance at the 5% significance level using chi-squared test. *n* = frequency, % = percentage.

### Adherence to the Mediterranean diet before and during the COVID-19 lockdown

**Fig 1:**
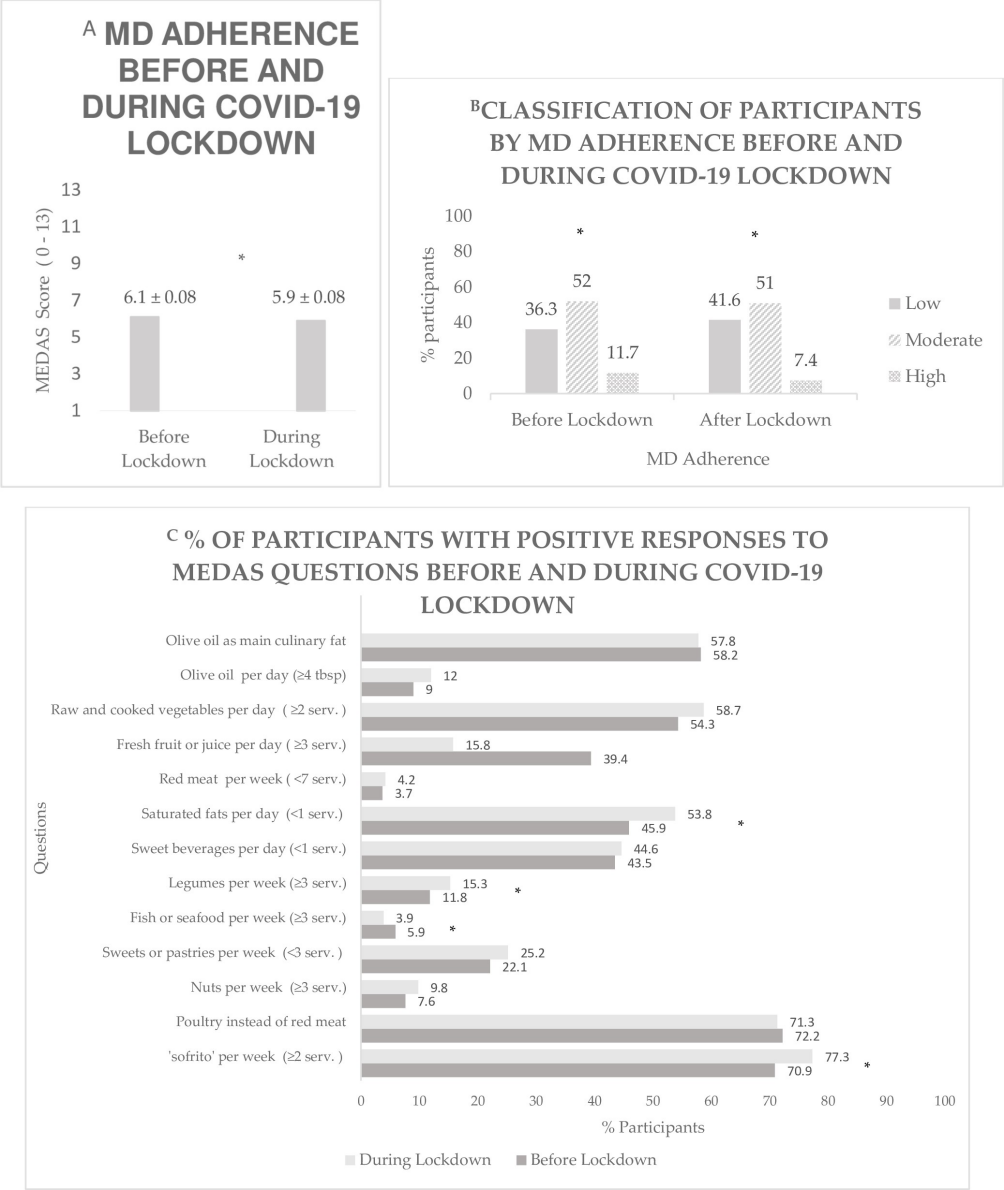
A Dietary Habits Before and During COVID-19 Lockdown: MD Adherence Score Before and During Lockdown. * indicates statistically significant differences in MD adherence score before and during lockdown, at the 5% significance level, using paired t tests. **B Dietary Habits Before and During COVID-19 Lockdown: Classification of Participants by MD Adherence (Low ≤ 5; Medium 6–8; High ≥ 9) Before and During Lockdown.** * indicates statistically significant differences in MD adherence (low, moderate, high) before and during lockdown, at the 5% significance level using a Bowker’s symmetry test. **C Dietary Habits Before and During COVID-19 Lockdown: Percentage of Participants with Positive Responses to MEDAS Questions Before and During the COVID-19 Lockdown.** Values expressed as mean ± standard deviation. * indicates statistically significant differences in positive responses to MEDAS questions before and during lockdown, at the 5% significance level, using a McNemar test for paired binary variables.

Fig 1A presents the score results of the adherence to the MD before and during the COVID-19 lockdown. There was a statistically significant reduction in MEDAS mean score during the studied period (before vs. during lockdown: 6.1 ± 0.08 vs. 5.9 ± 0.08), with a mean difference of -0.20 (95% CI = -0.31, -0.90; *P* < 0.001). As reported in Fig 1B, 36.3% of participants were classified as having low adherence (score ranged from 1–5), 52% of participants as moderate adherence (score ranged from 6–8), while 11.7% of participants as high adherence (score ranged from 9–11) to the MD before lockdown (p < 0.001). During lockdown, 41.6% of participants were classified as having low adherence (score ranged from 1–5), 51% as moderate adherence (score ranged from 6–8) and 7.4% as high adherence to the MD (score ranged from 9–11, p < 0.001).

The breakdown of the MEDAS components before and during the COVID-19 lockdown in Qatar are presented in Fig 1C. A statistically significant increase was seen in the percentage of participants who consumed four or more tablespoons of olive oil per day (9% vs. 12%; *p* < 0.001); two or more servings of raw or cooked vegetables per day (54.3% vs. 58.7%; *p* = 0.005); three or more servings of legumes per week (11.8% vs. 15.3%; *p* = 0.007) and two or more servings of ‘sofrito’ per week (70.9% vs. 77.3%; p < 0.001) when comparing before and during lockdown. On the other hand, the percentage of participants who consumed three or more servings of fresh fruit or fruit juice per day (39.4% vs. 15.8%; *p* < 0.001) and three or more servings of fish or seafood per week (5.9% vs. 3.9%; *p* = 0.035) significantly decreased during lockdown compared to before lockdown. Additionally, there was a significant increase in the percentage of participants who consumed less than one serving of fats (such as butter, margarine, ghee or cream) per day, when comparing before with during the lockdown (45.9% vs. 53.8%; *p* < 0.001).

### Physical activity before and during the COVID-19 lockdown

**Fig 2:**
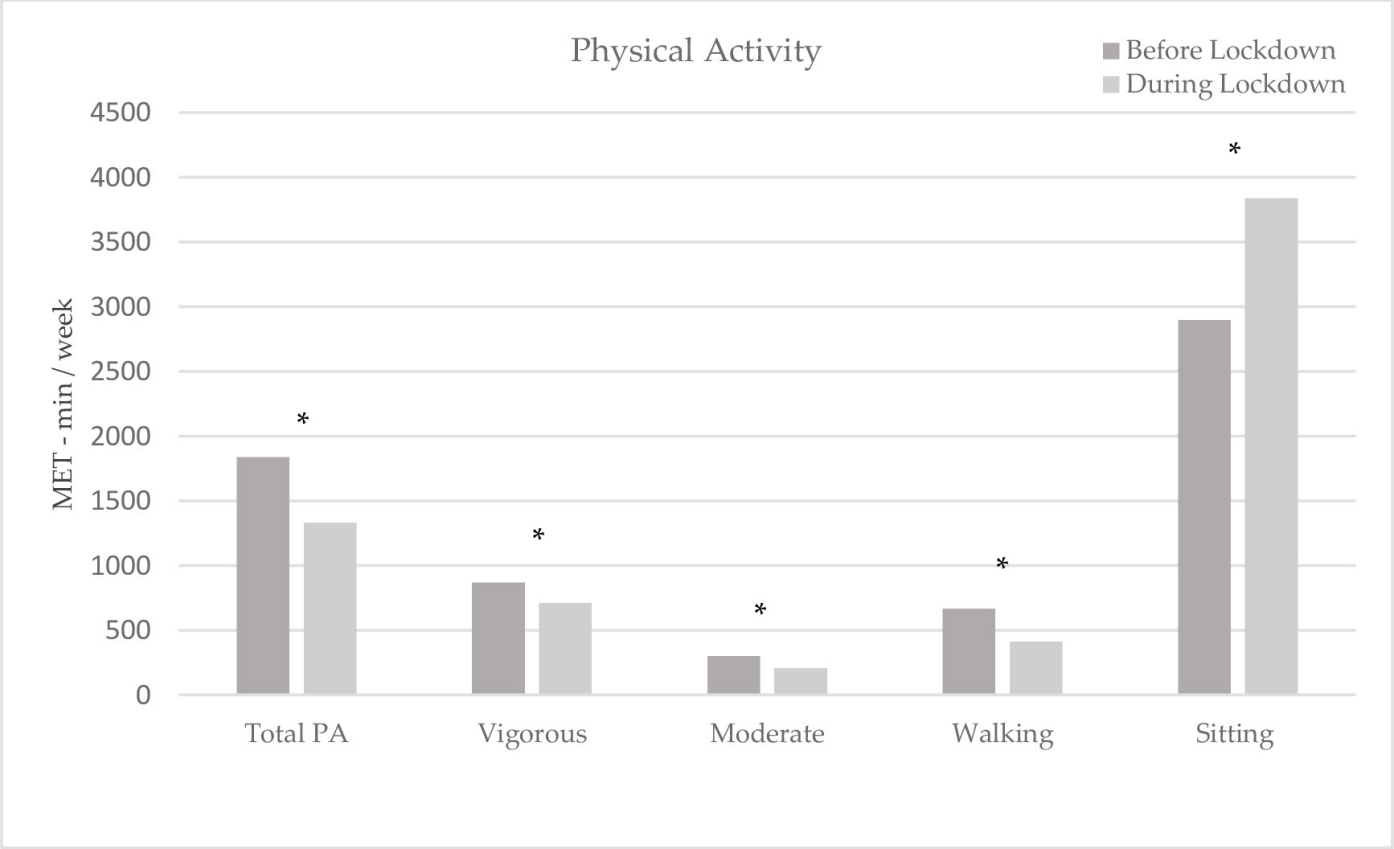
Comparison of PA before and during the COVID-19 lockdown. Total PA, vigorous and moderate PA, walking and sitting in MET-min/week, before and during the COVID-19 lockdown. Data expressed as mean values. * indicates statistically significant differences physical activity before and during lockdown, at the 5% significance level, using paired t tests.

Responses to the physical activity questionnaire completed before and during the COVID-19 lockdown are presented in Fig 2. The total physical activity was significantly reduced from 1836.7 ± 2087.9 MET-min/week before COVID-19 lockdown compared to 1330.5 ± 1663.7 during COVID-19 lockdown (*B* = -506.26; 95% CI = -678.60, -333.92; *p* < 0.001). Specifically, MET-min/week values of vigorous intensity activity (867.4 ± 1412.4 vs. 711.5 ± 1190.4; *B* = -155.95; 95% CI = -274.64, - 38.21; *p* = 0.010), moderate intensity activity (301.3 ± 677.0 vs. 208.3 ± 498.4; *B* = -93.04; 95% CI = -148.07, -38.01; *p* = 0.010) and walking (668.0 ± 838.4 vs. 410.7 ± 630.8; *B* = -257.27; 95% CI = -337.87, -176.67; *p* < 0.001) were significantly lower during lockdown compared to before lockdown. Sitting, on the other hand, was significantly increased during the COVID-19 lockdown, with mean MET-min/week values of 2896.4 ± 199.7 compared to 3837.3 ± 1181.2 before lockdown (*B* = 940.91, 95% CI = 831.9, 1049.90; *p* <0.001).

### Personality and adherence to the Mediterranean diet before and during the COVID-19 lockdown

An association between the different personality dimensions and MEDAS score was found in our study population (Table 2).

**Table 2:** Association between MD adherence and types of personality (n = 543) before and during the COVID-19 lockdown.

		Personality Type								
MD Adherence	Agreeableness		Extraversion		Conscientiousness		Neuroticism		Openness	
	Model 1	Model 2	Model 1	Model 2	Model 1	Model 2	Model1	Model 2	Model 1	Model 2
Before Lockdown	*Ref (5*.*89)*^a^	*Ref*	0.95 (0.20,1.69)	0.84 (0.04,1.64)	0.71 (0.25,1.16)	0.46 (-0.02,0.93)	-0.43 (-1.12,0.27)	-0.68 (-1.41,0.06)	0.22 (-0.15,0.59)	0.20 (-0.16,0.57)
P- value			*0.013	*0.039	*0.002	0.060	0.230	0.072	0.247	0.273
During Lockdown	*Ref (5*.*88)*^a^	*Ref*	0.33 (-0.55,1.21)	0.26 (-0.61,1,13)	0.47 (0.05,0.90)	0.24 (-0.20,0.68)	-0.34 (-1.11,0.42)	-0.53 (-1.35,0.28)	-0.07 (-0.43,0.28)	-0.10 (-0.47,0.26)
P- value			0.457	0.556	*0.027	0.289	0.379	0.198	0.689	0.578
Change in Score	*Ref (-0*.*01)*^a^	*Ref*	-0.61 (-1.18,-0.49)	-0.58 (-1.20,0.04)	-0.23 (0.48,0.02)	-0.22 (-0.47,0.04)	0.08 (-0.49,0.66)	0.14 (-0.45,0.74)	-0.29 (-0.56,-0.02)	-0.31 (0.58,-0.04)
P- value			*0.033	0.067	0.074	0.102	0.775	0.635	*0.036	*0.026

Model 1: Unadjusted

Model 2: Adjusted for gender, age, nationality, education, marital status, work status, BMI, smoking

Mean difference (95% CI) between each personality type and the personality type agreeableness (reference category)

* indicates statistical significance at the 5% significance level using linear regression.

^a^ indicates mean of reference category (agreeableness)

Before the COVID-19 lockdown, participants scoring highly in extraversion (B = 0.95; 95% CI = 0.20, 1.69; *p* = 0.013) and conscientiousness (B = 0.71; 95% CI = 0.25, 1.16; *p* = 0.002) had higher MEDAS scores than participants scoring highly in agreeableness (treated as the reference personality trait). After adjusting for confounders, the association remained robust for extraversion (B = 0.84; 95% CI = 0.04, 1.64; *p* = 0.039), whereas the association with conscientiousness decreased and became marginally non-significant (p = 0.060). During the COVID-19 lockdown, those with higher scores for conscientiousness had higher MEDAS scores compared to agreeableness (B = 0.47; 95% CI = 0.05, 0.90; *p* = 0.027). This association, however, was attenuated after adjusting for confounders in model 2. Concerning the change in MEDAS score, both extraversion (B = -0.61; 95%CI = -1.18, -0.49; *p* = 0.033) and openness (B = -0.29; 95% CI = -0.56, -0.02; *p* = 0.036) scores did not change as much compared to agreeableness before and during lockdown. After adjusting for confounders, however, the association remained robust only for those with high openness scores (B = -0.31; 95% CI = -0.58, -0.04; *p* = 0.026). The changes in MD scores for each category can be seen in S1 Table.

### Personality and physical activity before and during the COVID-19 lockdown

Associations of personality dimensions and PA before and during the COVID-19 lockdown are presented in Table 3. Among the personality dimensions examined, and using the agreeableness personality trait as the reference, those who scored high in openness were more likely to engage in all PA (*B* = 562.2; 95% CI = 62.7, 106.7; *p* = 0.027), including walking (*B* = 241.7; 95% CI = 29.4, 454.0; *p* = 0.026), before lockdown, even after adjusting for confounders. Those with high openness scores were also least likely to be sitting (*B* = -303.4; 95% CI = -590.0; -16.8; *p* = 0.038) when compared to those with high agreeableness scores before lockdown. During lockdown, this association became less apparent and did not reach statistical significance. Those with high neuroticism scores spent less time sitting when compared to those with high agreeableness scores. Although the specific comparison did not reach statistical significance before lockdown, it did during lockdown, after adjusting for confounders in model 2 (*B* = -619.5; 95% CI = -1215,-23.9; *p* = 0.042). Associations of other physical activity characteristics not discussed here are presented in S2 Table. Additionally, the PA MET/min-week values for each personality trait are shown in S3 Table.

**Table 3:** Physical activity characteristics by personality traits before and during the COVID-19 lockdown.

		Personality Type									
PA (MET values)		Agreeableness		Extraversion		Conscientiousness		Neuroticism		Openness	
		Model 1	Model2	Model 1	Model 2	Model 1	Model 2	Model 1	Model 2	Model 1	Model 2
All PA	Before Lockdown	*Ref* (1566.4)^a^	*Ref*	544.9 (-404.2, 1514)	499.5 (-513, 1512)	272.8 (-266.6, 812.2)	221.2 (-357.7, 800.1)	-222.7 (-1113.3, 668.1)	-228.5 (-137.4, 680.5)	481.1 (-3.9, 966.2)	562.2 (62.7, 1061.7)
	*P*-value			0.256	0.333	0.321	0.453	0.623	0.621	0.052*	0.027*
	During Lockdown	*Ref* (1114.5)^a^	*Ref*	559.5 (-110.8, 1230.6)	426.3 (-240.3, 1093)	295.7 (-143.2, 734.6)	187.3 (-288.2, 662.7)	193.2 (-624.6, 1011.2)	102.2 (-744.4, 948.8)	311.8 (-78.8, 702.3)	358.9 (-537, 771.5)
	*P*-value		0.102	0.209	0.186	0.439	0.642	0.813	0.117	0.088
Walking	Before Lockdown	*Ref* (584.0)^a^	*Ref*	-157.8 (340.8, 25.2)	-96.0 (-321.3, 129.3)	34.6 (-182.1, 251.3)	100.0 (-162.7, 362.8)	-188.0 (-427.2, 51.2)	-134.2 (-428.4, 160.0)	205.0 (458.8, 709.2)	241.7 (29.4, 454.0)
	*P*-value		0.091	0.403	0.754	0.455	0.123	0.370	0.040*	0.026*
	During Lockdown	*Ref* (311.3)^a^	*Ref*	139.7 (-56.4, 335.9)	117.3 (-99.5, 334.2)	146.1 (-27.1, 319.3)	74.5 (-95.5, 224.4)	14.9 (-197.3, 227.1)	-11.5 (-229.6, 206.2)	153.4 (11.8, 295.0)	149.2 (-11.3, 309.7)
	*P*-value		0.162	0.288	0.098	0.390	0.890	0.916	0.034*	0.068
Sitting	Before Lockdown	*Ref* (3017.6)^a^	*Ref*	-287.6 (-949, 373.7)	-322.0 (-1005.2, 361.2)	45.4 (-310.8, 407.5)	-86.0 (-470.6, 298.7)	-497.6 (-1092.8, 97.5)	-549.2 (-1177.4, 79.1)	-243.4 (-518.4, 31.7)	-303.4 (-590.0, -16.8)
	*P*-value		0.393	0.355	0.791	0.661	0.101	0.086	0.083	0.038*
	During Lockdown	*Ref* (3917.6)^a^	*Ref*	-347.6 (-1042.5, 347.2)	-373.2 (-1099.3, 352.9)	22 (-325.3, 369.2)	-118.0 (-499.8, 263.7)	-573.8 (-1191.8, 44.2)	-619.5 (-1215.2, - 23.9)	-126.3 (-402.9, 150.3)	-179.5 (-472.6, 113.6)
	*P*-value		0.326	0.313	0.901	0.544	0.069	*0*.042*	0.370	0.229

Model 1: Unadjusted

Model 2: Adjusted for gender, age, nationality, education, marital status, work status, BMI, smoking

Mean difference (95% CI) between each personality type and Agreeableness (reference category)

*indicates statistical significance at the 5% significance level using linear regression

^a^ indicates means of reference category (agreeableness)

## Discussion

To our knowledge, this is the first study to investigate the association between personality and lifestyle habits such as diet and exercise during the COVID-19 lockdown in Qatar. This adds to previously conducted research in other population groups which mainly focused on lifestyle without considering personality and provides evidence to further understand the association between lifestyle and personality during such unprecedented and difficult circumstances. Noticeably, there was a reduction in MD adherence during lockdown in the study population. Although lockdown was associated with an increase in olive oil, vegetables, legumes, sofrito and fat consumption, there was also a significant reduction in fresh fruit and fish/seafood consumption during lockdown. Total PA (vigorous and moderate activity) and walking were reduced during lockdown, while the time spent sitting was increased compared to before lockdown. Certain personality types such as extraversion had higher MD adherence before but not during lockdown. Openness was associated with no change in the MEDAS score during lockdown. Participants who scored high in openness were more likely to engage in PA (vigorous and moderate) and walking and less likely to be sitting before lockdown. Additionally, during lockdown, those who scored high in neuroticism were less likely to be sitting.

Concerning demographics, women scored highly in the agreeableness dimension, which was also reported in other studies in various cultures [31]. In contrast, however, we found that the majority of men scored high in the conscientiousness dimension, which contradicts some studies that reported women as having higher scores in this dimension [31]. This finding, however, is not consistent across various cultures [32], which could explain our findings. With regard to BMI, our finding of high agreeableness levels being associated with underweight or normal body weight is consistent with previous studies [33]. Those who were overweight or obese scored high in the openness dimension, and this contradicts other studies which reported that those who scored high in openness were less likely to be overweight or obese [33]. This could also be due to cultural differences.

Overall, our study found a significant but small reduction in the MD adherence score during the COVID-19 lockdown by an average of 0.2 points. This contrasts with other studies in Spain and Chile, where participants had adopted MD eating patterns during lockdown [34, 35]. As suggested by Di Renzo et al. [36], the MD could be one of the best food models for strengthening immunity and protecting against COVID-19. Therefore, reduced adherence to the MD could compromise nutritional quality, reduce the intake of foods high in antioxidant and anti-inflammatory properties, and as a result increase susceptibility to viral infections. Our findings on worsening eating habits, such as a decrease in fresh fruit and fish/seafood and an increase in fat consumption, were also similarly observed in other studies [37, 38]. A study on French adults reported that individuals bought fewer fresh products such as fruit due to limited shopping and a lack of access to their usual food shops, leading to the replacement of fruits with frozen vegetables [39]. Similarly, a study in Kuwait [40] also reported an overall significant reduction in fish and seafood consumption. The decrease in fresh fruit and seafood consumption in our findings was expected, since both fish and fruit markets in Qatar were closed during lockdown. It is also culturally preferred to consume fresh rather than frozen fish, therefore contributing to this reduction in consumption. On the other hand, there was an improvement in some dietary components in our study during lockdown, such as the consumption of more olive oil, vegetables, legumes and sofrito, similarly reported in Spanish [34] and Cypriot studies [41]. Similarly, another study looking at food behavior and consumption in Qatar found that 32.4% of respondents increased their consumption of vegetables during lockdown [3]. These findings could be explained by the increased consumption of locally produced legumes and vegetables due to food safety concerns and the uncertainty of the origins of imported food [3].

With regard to PA, all types (vigorous, moderate and walking) were significantly reduced during lockdown, while sitting was increased. These results were expected considering the country was under lockdown and there was no access to gyms or permission to freely leave the house. Our findings are similar to other studies that reported a significant reduction in PA due to the limitations during lockdown [10]. Similarly, Abed Alah et al. [8] and Hermassi et al. [9] both reported a reduction in all PA activities and an increase in daily sitting time in the Qatari population. Our findings, however, do contradict other studies that reported an increase or maintenance of physical activity during lockdown [11, 12].

With regards to personality and diet associations, our study did find that extraversion was associated with a higher MD adherence before lockdown. This is similar to another study by Mottus et al. [42], who reported that those with high scores of extraversion followed a Mediterranean style diet. However, our results suggest that no personality dimension was correlated with MD adherence during lockdown, after adjusting for confounders. This was not expected, as previous studies reported that those who score high in conscientiousness are known to be organized and therefore practice dietary restraint and tend to follow a healthy dietary pattern [43, 44]. Furthermore, the MEDAS score did not change during the lockdown for those with high scores for the openness personality dimension. This is not surprising, since high levels of openness have been associated with a Mediterranean style dietary pattern in previous studies [42, 45]. Openness has also been linked to resilience and ability to adapt to adverse life events [45], which could explain why there was no observed change in MEDAS score during the pandemic in our study.

In relation to personality and its association with PA, those with high scores for the openness personality dimension were associated with all PA (vigorous, moderate and walking), and spent less time sitting before the lockdown. However, there was no association with any personality and PA during the lockdown. This is contradictory to a recent study by Stephan et al. [21], that reported that those high in conscientiousness and agreeableness and low in neuroticism were associated with an increase in physical activity during the pandemic. Interestingly, though, in our study, those who scored high in neuroticism spent less time sitting compared to other personality dimensions, even after adjusting for confounders. Although evidence indicates that high neuroticism is associated with poor outcomes [46], typically stable personality traits change in response to distress [47]. As an example, a study during the COVID-19 outbreak in Canada reported changes in the five-factor model personality trait [48]. Neuroticism, for instance, decreased across six weeks of lockdown, indicating that feelings of anxiety and distress associated with this personality dimension could be attributed to the pandemic itself rather than one’s personality traits [48]. Furthermore, they reported acute personality changes related to isolation in those with conscientiousness, agreeableness, neuroticism and openness traits. Our findings similarly suggest acute changes and instability of the five factor personality traits due to the COVID-19 pandemic. It is likely that those who score high in the neuroticism dimension are less likely to spend their time sitting to avoid risky behavior out of worry and perceived fear of the pandemic. A few studies showed that those who score high in neuroticism tend to be physically active [49], vigilant about their health [50] and are compliant with policies that would increase their safety [51]. A study by Abdelrahman et al. [52] on Qatari residents looked at social distancing practices during the COVID-19 pandemic and found that neuroticism was positively correlated with adopting social distancing. Furthermore, Aschwanden et al. [16] concluded that those who are neurotic adopt health-promoting behaviors unexpected during normal circumstances possibly due to the anxiety and fear evoked by the pandemic.

Nevertheless, studies that have examined health behaviors and personality traits during the COVID-19 pandemic are scarce. A recent study on a Finnish population of women aged 47–55 years investigated the association between personality and changes in health behaviors and depressive symptoms during the pandemic [23]. They reported a decrease in alcohol consumption and changes to healthier eating habits during the pandemic in women with higher extraversion personality. On the other hand, women who scored high in neuroticism had both healthy and unhealthy eating habits [23]. This highlights that anxiety provoked maladaptive and vigilance behaviors, which can occur in the neuroticism personality dimension, and was similarly found in our study with regard to time spent sitting.

The above findings demonstrate that the COVID-19 lockdown had variable impacts on the health behaviors of individuals. Understanding the links between personality and health behaviors can potentially target future policies and interventions aiming to improve eating habits and physical activity in the context of responses to unprecedented situations, such as the COVID-19 lockdown. Although an individual’s personality may predict eating and physical activity behavior changes under normal circumstances [53], these associations were not evident during the lockdown, suggesting that personality may not be as strong a determinant during this period. These findings are thus useful in pointing out that healthy dietary and physical activity behaviors should be encouraged for all personality types during exceptional circumstances. Nevertheless, longitudinal assessments of longer duration and in different populations on how personality impacts health behaviors are required to further understand the impact over time and among different populations. In addition, future research should consider the cultural dimensions that are weaved into observed individual differences, measured by the different personality inventories.

This study presents some strengths and limitations. The main strength of this study was that it provided, for the first time, observational data on associations between diet, PA and personality dimensions during the COVID-19 lockdown in Qatar. Futhermore, this study used assessment tools (MEDAS and the IPAQ-SF) which are validated and widely used instruments for assessing MD adherence and PA habits, respectively. With regard to limitations, the cross-sectional design of the study does not allow us to draw conclusions on cause-and-effect relationships. Moreover, dietary and PA changes were self-reported, and therefore might be susceptible to recall bias based on the ‘before lockdown’ component. Thirdly, the study population had a high proportion of participants who were single, female students, aged 21–29 years with an undergraduate degree education level, thus limiting the generalizability of the findings. This was due to the oversampling of networks related to the non-random convenient sampling method, and as recruitment was conducted mostly through social media, it mainly targeted younger people. Although validated tools were used, online surveys have inherent limitations due to their subjective interpretations and perceptions. Lastly, at the time of conducting our study, there was no research on personality and thus no reason to believe that the COVID-19 lockdown would have any effect on personality traits. Since then, there is a study that does suggest that the pandemic could change certain personality traits [47]. Longitudinal studies with representative samples should be conducted to better understand the lasting effects of the COVID-19 lockdown and the restrictions put in place on dietary and PA habits, their changes over time and associations with the different personality dimensions.

## Conclusions

The present study provided, for the first time, information on eating habits and PA habits during the COVID-19 lockdown period in Qatar as well as associations with personality. Our results reveal a reduction in PA and changes in eating habits during the lockdown period. Personality types were associated with dietary habits and PA mainly before rather than during lockdown. Poor dietary habits, together with an unhealthy lifestyle, can cause serious health problems; therefore, adverse lifestyle changes during lockdown periods should be considered carefully. Although our study was conducted in Qatar, our findings may be generalized to other populations with similar demographic characteristics, due to the similarities of lockdown measures across the world. Future work will need to address whether there are long-term changes in diet and exercise habits and how these are associated with different personality dimensions in response to the ongoing COVID-19 pandemic. The findings should be used to develop appropriate personality-tailored lifestyle interventions to reduce unwarranted negative health effects during, but not restricted to, lockdown periods.

## Supporting information

S1 Table MEDAS mean score by personality types before and during COVID-19 lockdown. (PDF)

S2 Table Physical activity characteristics by personality traits before and during the COVID-19 lockdown. (PDF)

S3 Table Physical activity mean MET values by personality traits before and during COVID-19 lockdown. (PDF)
