# Supplementary material for: Impact of the COVID-19 lockdown on lifestyle behaviors and their association with personality among adults in Qatar: A cross-sectional study
Source: PLoS One. 2022 Nov 11;17(11):e0276426. doi: 10.1371/journal.pone.0276426 (PMC9651556; doi:10.1371/journal.pone.0276426)
Supplement: S1 Table — (PDF) [file pone.0276426.s001.pdf]

S1 Table. MEDAS Mean Score by Personality Types Before and During COVID-19 Lockdown.

MEDAS mean score	Agreeableness	Extraversion	Conscientiousness	Neuroticism	Openness
Before Lockdown	5.9	6.8	6.6	5.5	6.1
During Lockdown	5.9	6.2	6.4	5.5	5.8
Change in Score	-0.1	-0.6	-0.2	0.1	-0.3
