# Supplementary material for: Impact of the COVID-19 lockdown on lifestyle behaviors and their association with personality among adults in Qatar: A cross-sectional study
Source: PLoS One. 2022 Nov 11;17(11):e0276426. doi: 10.1371/journal.pone.0276426 (PMC9651556; doi:10.1371/journal.pone.0276426)
Supplement: S2 Table — (PDF) [file pone.0276426.s002.pdf]

Table S2: Physical Activity Characteristics by Personality Traits before and During the COVID-19 Lockdown

			**Personality Type**							
**PA (MET values)**		**Agreeableness**	**Extraversion**		**Conscientiousness**		**Neuroticism**		**Openness**	
			**Model 1**	**Model 2[a]**	**Model 1**	**Model 2[a]**	**Model 1**	**Model 2[a]**	**Model 1**	**Model 2[a]**
Vigorous Activity	Before Lockdown	*Ref* (732.4)	667.6 (-139.8, 1474.9)	572.2 (-297.6, 1442.0)	246.2 (-143.5, 635.9)	146.9 (-266.9, 560.7)	-74.0 (-758.0, 610.1)	-127.3 (-805.3,550.8)	159.4 (-156.2, 474.9)	175.1 (-148.8,499)
	P-value		0.105	0.197	0.215	0.486	0.832	0.712	0.321	0.288
	During Lockdown	*Ref* (626.9)	389.8 (-120.9, 900.4)	314.8 (-205.0, 834.7)	129.9 (1-87.1, 446.9)	128.8 (-233.5, 491.0)	-11.5 (-496.8,473.8)	-54.1 (-573.8,465.7)	110.2 (-171.6,392.0)	153.9 (-147.8,455.5)
	P-value		0.134	0.234	0.421	0.485	0.963	0.838	0.442	0.316
Moderate activity	Before Lockdown	*Ref* (249.9)	45.1 (-213.9,304.0)	23.3 (-244.1,290.7)	-8.0 (-158.5,142.4)	-25.7 (-184.1,132.7)	39.3 (-498.3,577.0)	33.0 (-508.4,574.4)	116.5 (-49.6,282.6)	145.4 (-20.4,311.3)
	P-value		0.732	0.864	0.916	0.750	0.886	0.905	0.169	0.085
	During Lockdown	*Ref* (176.3)	30.4 (-118.7,179.5)	-5.8 (-148.0,136.3)	19.7 (-106.2,145.6)	-16.0 (-148.7,116.8)	189.9 (-442.8,822.5)	167.9 (-469.4,805.2)	48.2 (-63.3,159.6)	55.8 (-56.2,167.8)
	P-value		0.689	0.936	0.758	0.813	0.555	0.605	0.396	0.328

Model 1: Unadjusted
Model 2: Adjusted for gender, age, nationality, education, marital status, work status, BMI, smoking
Mean difference (95% CI) between each personality type and agreeableness (reference category)
***** indicates statistical significance at the 5% significance level using linear regression, [a] indicates means of reference category (agreeableness)
