# Supplementary material for: Impact of the COVID-19 lockdown on lifestyle behaviors and their association with personality among adults in Qatar: A cross-sectional study
Source: PLoS One. 2022 Nov 11;17(11):e0276426. doi: 10.1371/journal.pone.0276426 (PMC9651556; doi:10.1371/journal.pone.0276426)
Supplement: S3 Table — (PDF) [file pone.0276426.s003.pdf]

S3 Table. Physical Activity Mean MET Values by Personality Traits before and During COVID-19 Lockdown.

	**PA mean values (MET)**	**Agreeableness**	**Extraversion**	**Conscientiousness**	**Neuroticism**	**Openness**
All PA	Before Lockdown	1566.4	2121.3	1839.2	1343.7	2047.5
	After Lockdown	1114.5	1674.3	1410.2	1307.7	1426.3
Moderate	Before Lockdown	249.9	295.0	241.9	289.2	366.4
	After Lockdown	176.3	206.7	196.0	366.2	224.5
Vigorous	Before Lockdown	732.4	1400.0	978.7	658.5	891.8
	After Lockdown	626.9	1016.7	756.8	615.4	737.1
Walking	Before Lockdown	426.3	426.3	618.6	396.0	789.3
	After Lockdown	451.0	451.0	457.4	326.2	464.7
Sitting	Before Lockdown	2730.0	2730.0	3066.0	2520.0	2774.3
	After Lockdown	3570.0	3570.0	3939.6	3343.8	3791.4
